# Mechanisms Governing Immunotherapy Resistance in Pancreatic Ductal Adenocarcinoma

**DOI:** 10.3389/fimmu.2020.613815

**Published:** 2021-01-28

**Authors:** Zoe C. Schmiechen, Ingunn M. Stromnes

**Affiliations:** ^1^Center for Immunology, University of Minnesota Medical School, Minneapolis, MN, United States; ^2^Department of Microbiology and Immunology, University of Minnesota Medical School, Minneapolis, MN, United States; ^3^Masonic Cancer Center, University of Minnesota Medical School, Minneapolis, MN, United States; ^4^Center for Genome Engineering, University of Minnesota Medical School, Minneapolis, MN, United States

**Keywords:** pancreatic cancer, PD-1, PD-L1, pancreatic ductal adenocarcinoma, T cell, exhaustion, immunosuppression, immunotherapy

## Abstract

Pancreatic ductal adenocarcinoma (PDA) is a lethal malignancy with an overall 5-year survival rate of 10%. Disease lethality is due to late diagnosis, early metastasis and resistance to therapy, including immunotherapy. PDA creates a robust fibroinflammatory tumor microenvironment that contributes to immunotherapy resistance. While previously considered an immune privileged site, evidence demonstrates that in some cases tumor antigen-specific T cells infiltrate and preferentially accumulate in PDA and are central to tumor cell clearance and long-term remission. Nonetheless, PDA can rapidly evade an adaptive immune response using a myriad of mechanisms. Mounting evidence indicates PDA interferes with T cell differentiation into potent cytolytic effector T cells *via* deficiencies in naive T cell priming, inducing T cell suppression or promoting T cell exhaustion. Mechanistic research indicates that immunotherapy combinations that change the suppressive tumor microenvironment while engaging antigen-specific T cells is required for treatment of advanced disease. This review focuses on recent advances in understanding mechanisms limiting T cell function and current strategies to overcome immunotherapy resistance in PDA.

## Introduction

Pancreatic ductal adenocarcinoma (PDA) is projected to be the 2^nd^ leading cause of cancer related deaths by the year 2030, and has a dismal 5 year survival rate of 10% (1). Disease incidence is on the rise and lethality is attributed to late diagnosis, early metastasis, and therapeutic resistance (2). The median overall survival of patients with metastatic disease is 3 to 6 months, and for patients with locally advanced disease is 8 to 12 months (3, 4). PDA is resectable in less than 20% of cases (5) and often surgery fails to cure (6, 7). Thus, there remains an urgent and unmet need to develop safe and effective therapies for PDA patient treatment.

Immune checkpoint blockade (ICB) is a promising immunotherapy transforming the standard of care for several advanced malignancies (8). ICBs are monoclonal antibodies that interfere with PD-1 and/or CTLA-4 inhibitory proteins expressed on the surface of T cells. Unfortunately, ICB rarely demonstrates clinical responses in PDA (9, 10). A recent phase 2 clinical trial combining anti-CTLA-4 and anti-PD-L1 exhibited an objective response rate of a mere 3.1% (11). Elucidating the reasons for ICB failure is an active area of investigation by our lab and others to inform the design of more effective immune-based treatments for PDA patients.

Mutant *Kras* is an oncogenic driver in 92% of PDA patients (12), and is sufficient to drive preinvasive disease in murine models (13). The genetically engineered *Kras*^G12D/+^;*Trp53*^R172H/+^;*p48*-Cre or *Pdx1*-Cre (*KPC*) autochthonous PDA mouse model recapitulates hallmark features of the human disease and has been informative in this regard. Similar to the human disease, *KPC* PDA originates from precursor histologically defined lesions that are called pancreatic intraepithelial neoplasms (PanINs) (14). At disease inception, these PanINs promote a fibroinflammatory and suppressive tumor microenvironment (TME) (15). The formation of PanIN lesions includes infiltration of suppressive tumor-associated macrophages (TAMs), myeloid-derived suppressor cells (MDSCs), and regulatory T cells (Tregs) which dominate the early immune response and persist to limit T cell activity. In addition to the cellular component, PanINs are surrounded by a dense extracellular matrix (ECM) that contains collagen and hyaluronan. Notably, myeloid-cell induced inflammation is critical for PDA development (16) and limits CD8 T cell anti-tumor responses (17). Moreover, mutant *Kras* is also required for the maintenance of advanced PDA by promoting the fibroinflammatory stroma and metabolic reprogramming to upregulate glycolytic genes and glucose uptake (18–20).

**Figure 1:**
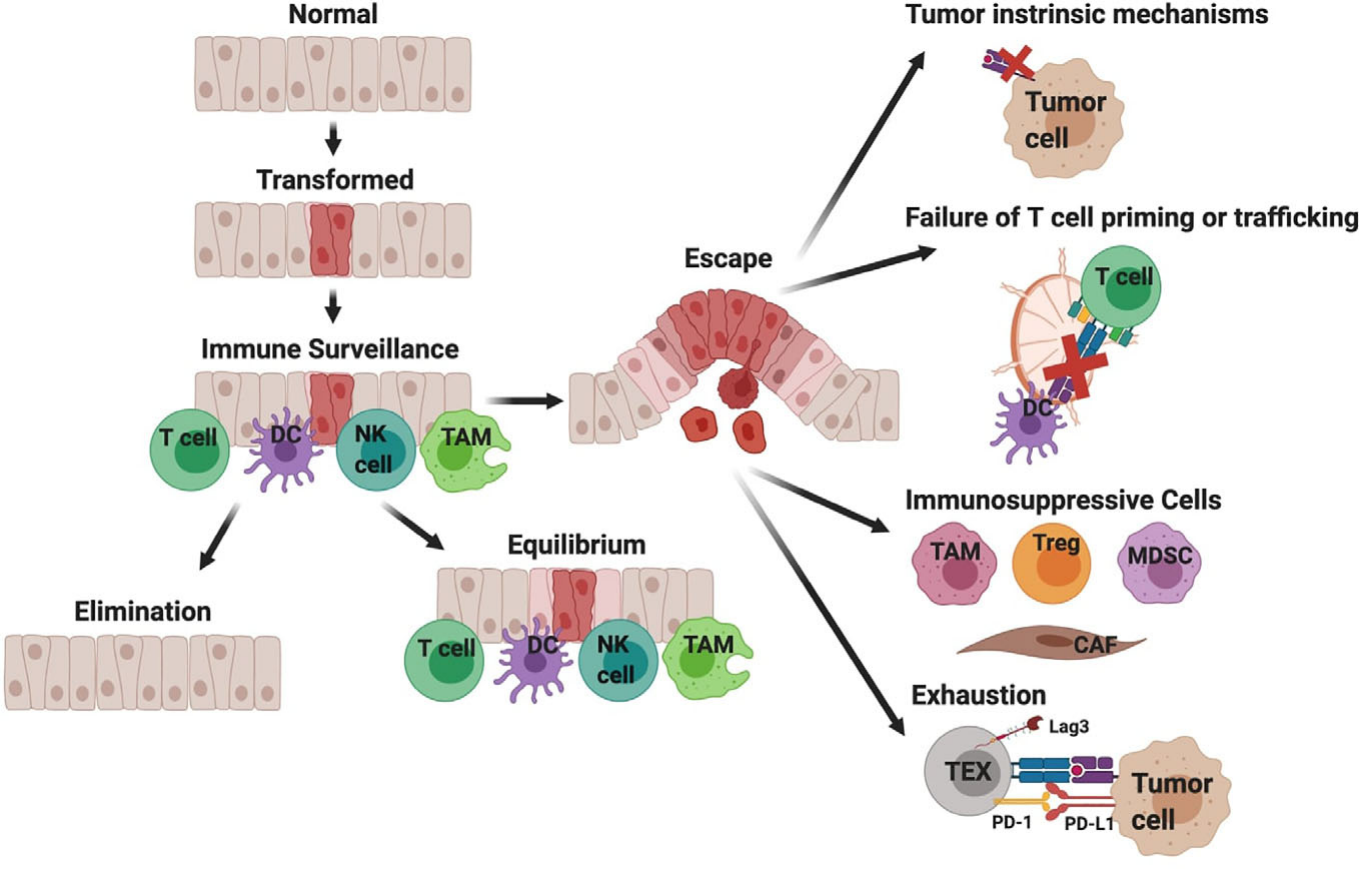
Simplified overview of immune surveillance and tumor evasion in pancreatic ductal adenocarcinoma (PDA). Immune surveillance is the process whereby the immune system surveys the body for malignant or infected cells. Components of immune surveillance include T cells, DCs, NK cells, and macrophages (TAM). Mutations in oncogene KRAS are a driver of PDA. The immune response to transformed tissue can either result in complete elimination of the malignancy, equilibrium to prevent further growth of the malignancy, or escape and development of clinically significant tumors. This figure provides a hypothesized sequence of immune evasion, but this process is likely not linear and instead a dynamic progression. Cancer cells can escape T cell recognition by losing target antigen expression and/or developing defects in antigen processing and presentation. Additionally, defects in T cell priming or trafficking to tumors may limit antigen-specific T cell responses and can be attributed to insufficient mature DCs. When antigen-specific T cells successfully infiltrate tumors, their function may be limited by immunosuppressive cytokines produced by macrophages (TAM, tumor-associated macrophage), regulatory T cells (Treg), myeloid-derived suppressor cells (MDSC), or cancer associated fibroblasts (CAF). Lastly chronic T cell receptor signaling drives T cell exhaustion, resulting in reduced effector function.

A hallmark of many cancers including PDA is immune evasion. When there is sufficient immune pressure by tumor-antigen-specific T cells, tumor variants may emerge that are defective in target antigen expression and/or antigen processing and presentation (21–24). Tumors that retain antigen presentation can still avoid T cell recognition by restricting antigen presenting cell differentiation, excluding T cells from tumor nests, immunosuppression, or induction of an altered T cell differentiation state referred to as T cell exhaustion (**Figure 1**) (25, 26). The particular mechanism(s) driving ICB resistance will impact therapeutic strategies to overcome it. We posit that there are similar mechanisms among multiple patient tumors, yet the hierarchy may depend upon the extent that tumor antigen-specific T cells are engaged. The goal of this review is to discuss major ICB resistance mechanisms and to highlight combination strategies to transform the TME to engage the anti-tumor T cells.

## Tumor Antigen-Specific T Cells Infiltrate PDA and Are Present in a Subset of Patient Tumors

T cells respond and mediate the anti-tumor effects of most immunotherapies. T cells express T cell receptors (TCRs) that specifically bind peptide:MHC complexes expressed on the cell surface of neighboring cells. During T cell development, most T cells strongly reactive to self-antigens are deleted in the thymus or tolerized in the periphery resulting in a T cell repertoire that is largely tolerant to self-antigens and reactive to foreign antigens (27). The number of nonsynonymous mutations, *e.g.*, tumor mutational burden (TMB), correlates with clinical responses following ICB (28, 29). A high TMB increases the likelihood that a particular mutation will code for a neoepitope, which is a novel tumor-specific antigen recognized by rare antigen-specific T cells. PDAs typically harbor ~25–60 somatic coding mutations (30–32), logs lower than some ICB-responsive cancers such as melanoma (33). A minor fraction of PDAs (<1%) have an abnormally high TMB due to genetic defects in mismatch-repair genes (34). A subset of these patients (5/8) respond to PD-1 blockade, yet even in this setting, responses are often not durable (24, 34). In a long-term PDA survivor cohort, neoepitope similarity to microbial epitopes, rather than neoepitope quantity, correlated with overall patient survival (30). CD8 T cell production of effector molecules IFNγ and Granzyme B are also elevated in long-term survivors (35) and survival is longer when CD8 T cells are proximal to cancer cells (36). Although still ongoing, interim analysis of a combination of a CD40 agonist, anti-PD-1, and standard of care chemotherapy (gemcitabine + abraxane) induced objective responses in over 50% of advanced PDA patients (37). As most preclinical animal studies indicate that CD40 agonist (38–41) or anti-PD-L1 (23) requires T cells for antitumor activity, the apparent success of this ongoing clinical trial supports that endogenous tumor-reactive T cells are present in a subset of PDA patients, despite a relatively low TMB, and can be beneficial for treating advanced disease. Much work remains to determine the independent contributions of CD4 and CD8 T cells, the antigen-specificity of such T cells, and the mechanisms underlying tumor clearance.

**Figure 2:**
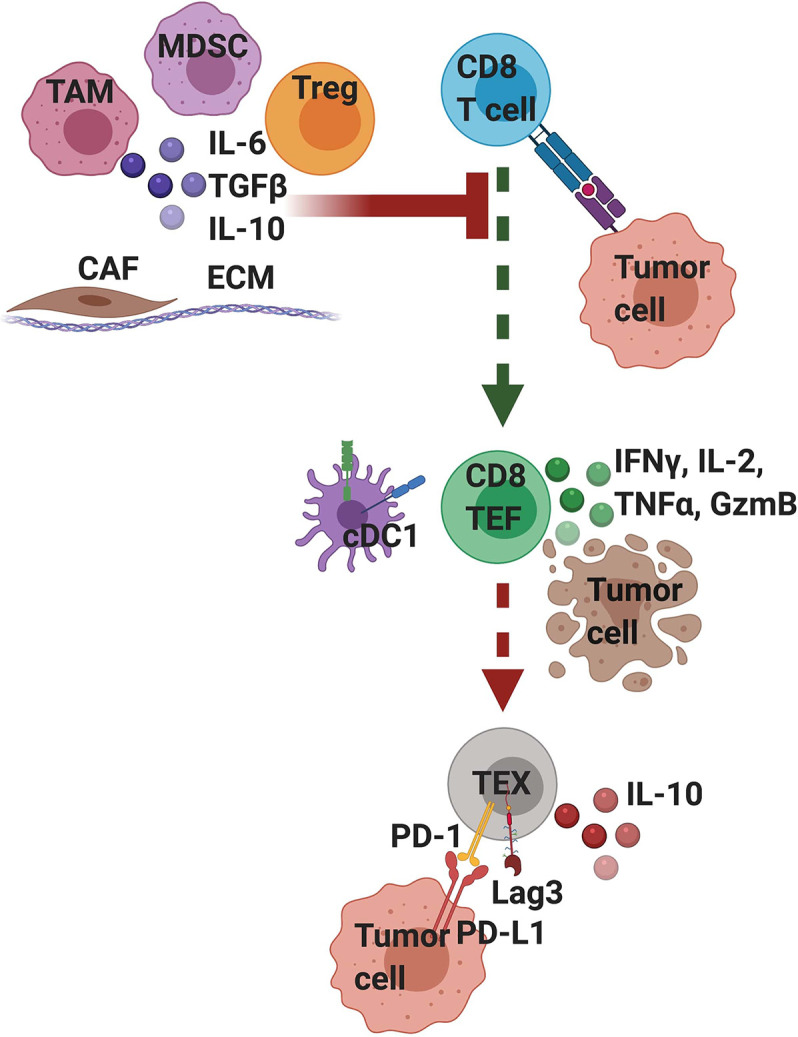
T cell intrinsic and extrinsic factors influence T cell differentiation and functionality in pancreatic cancer. Tumor-infiltrating T cells that strongly recognize tumor antigen mediate transient anti-tumor activity but if the tumor is not cleared, differentiate into exhausted (TEX) T cells, which is driven by persistent T cell receptor (TCR) signaling. Exhausted T cells are often characterized by their expression of PD-1 and Lag3 and are hypofunctional. Tumor cells and other stromal cells can express ligands for these inhibitory receptors, such as PD-L1, a ligand for PD- 1. Signaling through these inhibitory receptors interferes with T cell function and differentiation state. Moreover, exhausted T cells may participate in their own suppression by producing IL-10. A variety of extrinsic cells and factors enriched in the suppressive tumor microenvironment can interfere with TCR signaling and activation. These include tumor associated macrophages (TAM), myeloid-derived suppressor cells (MDSC), regulatory T cells (Tregs), and cancer associated fibroblasts (CAF) all embedded within a dense extracellular matrix (ECM). Immunosuppressive cells secrete cytokines including IL-10 and TGFβ that can suppress T cell activation and TCR signaling. Mechanistically, extrinsic suppression and TCR-driven exhaustion are distinct processes that converge to suppress tumor immunity. We posit that targeting immunosuppression may lead to only transient anti-tumor immune responses because if the tumor is not cleared; T cells will become exhausted. Thus, PDA likely requires combination immunotherapies that target both T cell extrinsic immunosuppression and T cell intrinsic exhaustion.

Historically, PDA has been considered immunologically “cold” as few T cells were found infiltrating tumor nests (42–44). In both the *KPC* mouse model and humans, tumor cells are surrounded by a robust fibroinflammatory stroma comprised of cancer-associated fibroblasts (CAFs), TAMs, Tregs, MDSCs, and rare endothelial cells embedded within a complex extracellular matrix (ECM) (**Figure 2**). In PDA patients, when CD8 T cells are present they are often contained within this stroma and often not directly contacting tumors cells (45, 46). T cell exclusion from tumor nests has been hypothesized to be due to “stromal trapping”, although mechanistically this process is ill-defined in patients (47, 48). Our *in situ* analysis of resected human PDAs identified marked heterogeneity among the number of T cells among independent tumor samples (45). A proportion of tumors contained few immune cells, a fraction contained abundant T cells within stroma, and a minor fraction of tumors contained abundant T cells within tumor cell nests. These results are consistent with a putative immunogenic subset based on transcriptional profiling (12) and indicate that T cells are present in a fraction of human tumors.

While murine models of PDA generally recapitulate the immune response seen in human PDA patients, there is a distinction with respect to in T cell infiltration. Although tumors from mouse models exhibit a similar T localization pattern within the stroma rather than direct contact with tumor cells (49, 50), murine models have few T cells in general, without the clear presence of a neoantigen or T cell engagement through immunotherapy (23, 50, 51). The increased heterogeneity in the T cell infiltrate in human compared to murine PDA models has spurred the recent development of mouse models to account for this variability (23, 52). Heterogeneity in T cell infiltration has been reported in murine tumors models in some cases and attributed to differences in tumor intrinsic genetic events (52) or antigenicity (23).

Contrasting with endogenous T cells of unknown antigen specificity that are excluded from PDA, we showed that T cells engineered to express a high affinity mesothelin (Msln)-specific TCR preferentially accumulate in tumors of *KPC* mice (53). Msln is a self/tumor antigen overexpressed by over 90% of PDAs (53, 54) and due to its low level of expression in normal tissues, is a target for immunotherapy. Thus, antigen-specific effector T cells can overcome stromal barriers and prolong animal survival.

Endogenous T cells specific to native antigens in *KPC* PDA have not been identified. There are few reported somatic mutations in *KPC* tumors (55) and as it is a spontaneous model, immunogenic epitopes, when present, will likely be different among individual *KPC* animals making it challenging to track a reproducible population of antigen-specific T cells. Therefore, we and others have developed tools to study model antigen-specific T cells. Tumor expression of ovalbumin (OVA) in *KPC* autochthonous mice unexpectedly was reported to accelerate tumor progression (56). In contrast, OVA-expressing *KPC* cell lines implanted subcutaneously or orthotopically are readily rejected in B6 animals (55) and can prompt outgrowth of OVA-loss variants (23). We developed a *KPC* orthotopic tumor model by expressing a click beetle red (CB) luciferase in a *KPC* cell line to monitor tumor growth in real time (23). Unlike the parental *KPC* CB-negative line that is refractory to anti-PD-1 or anti-PD-L1, CB+ tumors transiently respond to anti-PD-L1. We identified an immunodominant epitope CB_101-109_:H-2D^b^, generated a fluorescently-labeled peptide-MHC tetramer and showed that endogenous tumor antigen-specific T cells also preferentially accumulate in PDA (23). This is consistent with our prior study using engineered T cells (53), and contrasts with other studies demonstrating T cells of unknown specificity are excluded from the pancreatic TME (50, 51). As reports often evaluate polyclonal T cells without regard for antigen-specificity, and the polyclonal T cell population is remarkably diverse, the biology of rare tumor antigen-specific T cells can be lost when studying bulk T cells (57).

## CD8 T Cell Exhaustion Is Driven by Persistent TCR Signaling

T cell exhaustion was initially identified in models of chronic viral infection, but the full extent to which these findings translate to PDA, remains to be fully investigated. Exhausted T cells are an epigenetically regulated T cell differentiation state that occurs in settings of chronic antigen, is driven by persistent TCR signaling (58–60), and results in defective T cell effector functions (**Figure 2**). TCR signaling drives expression of the transcription factor Tox, which is required for the survival and persistence of exhausted T cells during chronic LCMV infection as well as in animal models of melanoma and liver cancer (61–63). In PDA animal models, we identified that either engineered or endogenous intratumoral tumor antigen-specific T cells also become defective in their production of IFNγ, TNFα and expression of cytolytic molecules including Granzyme B following specific peptide recognition (23, 53). In contrast, T cells with identical peptide specificity remained largely functional in the spleens within the same tumor-bearing animals, indicating that the tumor and/or the TME drive T cell functional loss. Tox is elevated in both functional effector splenic CD8 T cells as well as intratumoral exhausted CD8 T cells (64). Early during tumor growth, intratumoral tumor-specific T cells co-express both Tox and Granzyme B and produce cytokines following antigen encounter. Over time, however, persisting intratumoral T cells maintain Tox while losing Granzyme B and cytokine production following antigen encounter.

As both PD-1 (23) and Tox appear insufficient to identify exhausted T cells in PDA, we pursued further analysis of these populations. T cell expression of Klrg1 and Lag3 may distinguish these populations in murine PDA (23). Klrg1 is expressed by the majority of tumor-specific T cells in the spleens of tumor-bearing mice. In contrast, it is largely absent on T cells infiltrating PDA. Most Lag3+Klrg1- T cells were exhausted whereas Lag3-Klrg1+ T cells were enriched intratumorally during a productive immunotherapy responses (23, 64) and highly functional. Klrg1 is expressed on NK cells (65) and effector CD8 T cells during acute viral infection (66, 67). It is also expressed by both short-lived (68) or long-lived effector CD8 T cells (69) and can be downregulated during memory T cell transition (70). An inverse relationship between Tox and Klrg1 expression in CD8 T cells during viral infection has been reported (61, 71), and Klrg1 expression correlates with T-bet levels (66). Thus, while further studies are warranted, especially in human samples, Klrg1 may be an intratumoral biomarker for functional antigen-specific T cells.

In a subset of resected human PDAs, intratumoral T cells were enriched for expression of markers of recent or prolonged TCR signaling including 4-1BB and PD-1 (45) suggesting specific antigen recognition. A large fraction of PD-1+ T cells co-expressed putative exhaustion markers Lag3 and Tim-3, similar to exhausted T cells in murine models (23, 53). In the other fraction of tumors, T cells exhibited an effector or effector memory phenotype yet did not express markers indicative of recent antigen encounter. These data suggest that T cells are not specific to tumor antigens, antigen presentation is limiting, and/or immunosuppression is dominant in this fraction (45). Single cell sequencing of human and mouse tumors support an exhausted T cell phenotype based on enrichment of *LAG3* and *EOMES (*72). Further, patients with tumors that expressed high levels of multiple coinhibitory genes (*e.g.*, *LAG3, CTLA4*, and *HAVCR2*) had decreased survival compared to patients that expressed low inhibitory gene levels (47). However, many of these markers are also expressed by Tregs, and this data does not definitively identify T cell exhaustion.

The capacity of T cells to self-renew, *e.g.*, stemness, is an emerging concept of cell-based therapy and *Tcf7* appears to mark retention of T cell stemness (73). A subpopulation of exhausted T cells that expressed the transcription factor *Tcf7* (protein is Tcf1) and intermediate levels of PD-1 proliferate and differentiate into effector T cells following PD-1 blockade during chronic viral infection and melanoma, as opposed to PD1^high^Tcf1- terminally exhausted T cells which fail to proliferate following ICB (74–77). Thus, PD-1 may prevent Tcf1+ T cell differentiation into cytolytic effector T cells (78). As there are many differences between a disseminated chronic viral infection and PDA, we are interested if a similar T cells progenitor subset is present in this malignancy. As *Tcf7* is a Tox-responsive gene (79), Tox may promote exhausted T cell survival and persistence during chronic antigen settings *via Tcf7*. Due to the many differences such as the cytokine milieu, costimulatory/co-inhibitory markers, and antigen presenting cells in PDA, progenitor CD8 T cells may not be found in all situations of chronic antigen stimulation, which may partially explain why some malignancies fail to respond to PD-1/PD-L1 blockade.

We recently identified that intratumoral PD-1+Lag3+Tox+ exhausted tumor antigen-specific T cells not only lose functionality (*e.g.*, IFNγ, TNFα), they progressively express IL-10 specifically in the TME (64). As IL-10 is a regulatory cytokine (80), these data suggest that the TME instructs antigen-specific T cells to actually participate in immune suppression. The T cell intrinsic role of autocrine and paracrine IL-10 on the program of T cell exhaustion and overall antitumor activity of immune based therapies is of current interest. Evaluation of cytokines within the TME point toward IL-27. IL-27 receptor signaling can induce IL-10 (64) and promote a T cell exhaustion program (81). In orthotopic tumor bearing mice, intratumoral myeloid cells including granulocytes, TAMs, and dendritic cells (DC) upregulate IL-27 during tumorigenesis (64). Moreover, *Il27ra*^-/-^ mice had delayed tumor growth and a lower frequency of intratumoral antigen-specific T cells expressing Tox (64). Overall the data suggest that if endogenous tumor-specific T cells are engaged sufficiently in PDA, IL-27 in combination with chronic TCR signaling promotes tumor growth by inducing T cell exhaustion.

## Inflammatory Cytokines Bias CD4 Th17 Differentiation at the Expense of Th1

Antigen-specific CD4 T cells have not been assessed in PDA animal models or humans. Several murine studies have assessed polyclonal CD4 T cells and shown a role in PDA progression and immunotherapy response (40, 41, 82, 83). CD4 T cells express elevated PD-L1 and PD-L1 ligation promotes Th17 at the expense of Th1 polarization (84). Cytokines including IL-27, IL-6, TGFβ, and IL-23 (85) are elevated in PDA and promote Th17 differentiation (56, 86). TAM production of IL-10 also promotes Th2, Th17 or Treg differentiation (87). Pancreatic stellate cell production of IL-6 promotes Th17 differentiation in human PBMCs while decreasing overall T cell proliferation (88). Depletion of immunosuppressive TAMs (89) or genetic ablation of IL-35 or IL-10 (90) increased Th1 cytokine production. Combination of anti-IL-6 plus anti-PD-L1 reduced tumor growth and prolonged mouse survival (91). Mechanistically, this therapy was dependent on CD8 T cells which associated with increased intratumoral Th1 frequency (91). Taken together, the cytokine milieu in PDA biases CD4 T cells away from Th1 and toward Th2, Th17 and Treg which is beneficial for tumor growth.

## NK Cells Are Excluded From the Pancreatic TME Yet Have Therapeutic Potential

Often tumors can escape from T cell immune surveillance *via* selection of MHC class I loss variants. Natural killer (NK) cells can lyse MHC class I low cancer cells (92). Low frequencies of NK cells are typically found in tumors, despite being elevated in the peripheral blood of PDA patients (83, 93). Low intratumoral NK cell frequency in PDA has been attributed to tumor mutant *Kras* and *Myc* signaling repressing expression of the type 1 interferon pathway (94). Compared to healthy donors, NK cells from PDA patients have reduced expression of CXCR2, which is expressed on CD56^dim^ cytotoxic NK cells (95), thereby preventing intratumoral NK cell trafficking (93). Circulating NK cells in PDA patients are impaired in cytotoxic degranulation and have reduced IFNγ production, and this impairment was both progressive with disease stage and associated with poor survival (96). Circulating NK cells from PDA patients exhibited reduced expression of NKp30 compared with healthy donors, suggesting decreased cytotoxicity, as CD107a and NKp30 expression were correlated (96). In another study, pancreatic stellate cells reduced NK cell Granzyme B and IFNγ expression, whereas NK cells in the peripheral blood maintained these functions (97).

Immunotherapies improving NK cell infiltration into PDA can induce tumor regression in preclinical models. Type 1 interferon signaling induces intratumoral macrophages to produce CXCL13 and recruit CXCR5+ NK cells (94). A cleavable antibody specifically targeting mesothelin+ tumor cells and containing the chemoattractant CXCL16, which binds CXCR6, improved NK cell infiltration after adoptive transfer, which reduced tumor burden and prolonged survival in orthotopic or metastatic PDA murine models (98). *Ex vivo* stimulation and expansion of NK cells promoted NK cell-mediated tumor cell lysis *in vitro* and reduced tumor volume after transfer *in vivo* (93). Together, these data suggest NK cells may be useful for targeting PDA given sufficient numbers and trafficking signals.

## Tumor Cell-Intrinsic Immune Suppressive Mechanisms

Genetic mutations in tumor cells alter cell signaling and subsequently the stromal cells surrounding the tumor. These changes impact the ability of T cells to accumulate and function in PDA. This process was initially identified in melanoma (99) in which tumor β-catenin levels interfered with DC and T cell intratumoral accumulation. PDA is particularly high for β-catenin (100). Other mechanisms often operate through altered cytokines and chemokines. Elevated *Myc* in tumor cells increases CXCL1 which recruits CXCR2+ suppressive myeloid cells (52). Pancreatic tumor cell production of inflammatory IL-1β activates CAFs to produce IL-6, CCL2 and CCL5 that polarize immunosuppressive TAMs (101). IL-1β stimulation of tumor cells upregulate CXCL13 expression and recruit PD-L1+ regulatory B cells (Bregs) (102). IL-1β blockade combined with anti-PD-1 reduced orthotopic tumor weight and improved CD8 T cell infiltration (101). Knockdown of IL-1β decreased tumor growth and reversed TAM immune suppression while increasing the frequency of polyclonal CD8 T cells and their production of IFNγ and Granzyme B (101, 102). CAF production of CXCL12 also interferes with CD8 T cell infiltration (50, 101), supporting a stromal trapping mechanism. Finally, tumor cells produce Csf1 and IL-34 which recruit immunosuppressive Csf1r+ TAMs that produce IL-6, IL-10, and TGFβ (103, 104), all factors that promote a cycle of immune suppression.

A major tumor-cell intrinsic mechanisms of PDA immune evasion is the disruption of DC homeostasis. A specialized subset of conventional DCs (cDC1s) are efficient at cross-presenting tumor-associated antigen and appear essential for priming tumor-specific T cells (105). cDC1s expand and are necessary participants in mediating tumor control following multiple immunotherapies in PDA mouse models (41, 52, 103, 106). However, there is a scarcity of a cDC1s in the TME, and the DCs that are present have reduced expression of co-stimulatory and maturation markers CD40, CD80, CD86, and MHC class II (56, 107, 108). Tumor-secretion of G-CSF, promotes granulocytes and monocyte differentiation at the detriment of cDC1s (107). IL-6 neutralization in *KPC* mice increased frequency of cDC1s, which was attributed to reduced apoptosis of cDC1s (108). cDC1 enrichment, either measured by frequency in peripheral blood (107) or gene expression in tumor samples (109), is associated with a better prognosis and immunological response. CD8 T cell infiltration correlates with cDC1 frequency in PDA (52). Thus, once tumor-specific T cells are engaged within the TME, they may produce factors such as GM-CSF (110), Xcl1 (111), and Flt3L (112) that promote DC recruitment.

## Tumor Cell-Extrinsic Immune Suppressive Mechanisms

### Tumor-Associated Macrophages

Pancreas macrophages are derived from either circulating monocytes or from the embryo during development. Such embryonic-derived tissue resident macrophages are MHC class II low and can promote fibrosis, while monocyte-derived TAMs can be express high levels of MHC class II and promote anti-tumor T cells (113). Tissue resident macrophages are present in autochthonous *KPC* tumors and orthotopic tumors yet are absent from subcutaneous tumors, underscoring the importance of studying TAM biology within the pancreas (113). TAMs accumulate in PDA and are generally polarized to the immunosuppressive phenotype defined by Csf1R, CD206, and IL-10 expression along with reduced expression of MHC class II and Ly6C (89, 104, 113, 114). While PD-L1 is expressed on a subset of TAMs (45, 115), approximately 50% of intratumoral CD8 T cells also express PD-L1 in both human and murine PDA (84). PD-L1 on T cells ligates PD-1 on macrophages thereby inducing STAT6 signaling, suppressive TAM polarization, and immune tolerance (84). Tumor derived Galectin 9 ligation of Dectin 1 also induces suppressive TAM polarization and promotes polyclonal T cell tolerance (82, 116). Gas6 production by both CAFs and TAMs promote epithelial-mesenchymal transition (EMT) and tumor metastasis, while simultaneously interfering with NK cell proliferation and activation (117). As PDA metastases progress, macrophages are rendered progressively immunosuppressive and suppress CD8 T cell proliferation *via* production of granulin (118). Microbes that accumulate in PDA polarize immunosuppressive macrophages *via* TLR signaling and antibiotics reduced tumor weight and improved anti-PD1 therapy (87).

In contrast, anti-tumor macrophages have higher expression of Ly6C and MHC class II (114). In *KPC* tumors, low CD8 T cells correlated with local and systemic increase in CD11b+ myeloid cells and MDSCs (52). However, T cell infiltration positively correlated with TAMs and CD8+ T cells co-localized with TAMs within the tumor stroma in human samples (45), suggesting potentially a T cell supportive role for TAMs.

### Cancer-Associated Fibroblasts

CAFs synthesize collagen and hyaluronan and coordinate ECM signaling, thereby promoting fibroinflammation (72). There are three CAF subsets identified including αSMA+ myofibroblast CAFs that promote stromal remodeling (myCAFs), inflammatory CAFs that have a secretory phenotype and produce IL-6 (iCAFs), and MHC class II+ CAFs that present antigen (apCAFs) (72, 119). myCAFs are generated by TGFβ-signaling and iCAFs by IL-1 signaling (120). After co-culture with tumor organoids, pancreatic stellate cells (PSCs) differentiate into CAFs subtypes (119). CAFs produce IL-6, which is elevated in human and murine PDA serum (88, 91, 108). IL-6 production can promote PDA metastasis to the liver by acting on hepatocytes (121). *In vitro* co-cultures suggest that human αSMA+ CAFs inhibit T cell function *via* PGE2 and promote T cell co-inhibitory receptor expression (46). Notably, CAF depletion through genetic ablation or a CXCR4 inhibitor reduced tumor volume in autochthonous *KPC* mice, and synergized with PD-L1 blockade (50). A CXCR4 inhibitor with PD-1 blockade increased tumor cell apoptosis and CD8 T cell clonal expansion and effector function in a human PDA tumor slice model (48). Cancer cell intrinsic FAK signaling contributes to the dense fibrotic and immunosuppressive TME of PDA by promoting CAF accumulation (122). Pharmacological CAF inhibition in KPC mice significantly decreased PDA fibrosis, as seen by decreased collagen deposition and numbers of αSMA+ fibroblasts (122). However, CAFs may also keep tumors in check as depletion can promote more aggressive PDA in animal models (123, 124). We speculate that how CAFs are targeted, as well as the extent that antigen-specific T cells are engaged, may determine the outcome following CAF targeting.

### Myeloid-Derived Suppressor Cells

Myeloid-derived-suppressor-cells (MDSCs) are immature myeloid cells enriched in animal models of PDA (15, 51, 87, 101, 125–127). Both granulocytic and/or monocytic MDSCs suppress naïve T cell proliferation and induce T cell apoptosis (2), which is mediated by ROS and Arg1 production (126). Tumor cell production of GM-CSF and G-CSF (15, 125, 128), microbial signaling through TLR5 (87), and tumor cell production of IL-1β (101) promote MDSC expansion and their suppressive phenotype. The frequency and number of intratumoral CD8 T cells and MDSC negatively correlate in response to therapeutic autophagy inhibition (106), neoadjuvant therapy (129), and at baseline in *KPC* (52) and *KC* tumors (51). MDSC depletion with anti-Ly6G increased CD8 T cell infiltration in autochthonous PDA (15). MDSC depletion with anti-Gr-1 also reduced intratumoral Tregs and Tregs promoted MDSC survival (126), suggesting that MDSCs and Tregs sustain each other. Lastly, CXCR2 signaling in MDSCs and neutrophils plays a key role in establishing and maintaining the metastatic niche (130). Treatment of *KPC* mice with a CXCR2 inhibitor, reduced metastasis and increased survival (130). Moreover, genetic deletion or inhibition of CXCR2 increased T cell infiltration of PDA in *KPC* mice (130) or mice bearing subcutaneous tumors (131).

### Foxp3+ Regulatory T Cells

Tregs restrict T cell cytotoxicity and are barriers to the anti-tumor response in PDA. Tregs are enriched in both human and murine PDA, and progressively increase in frequency from early stages of tumor development (45, 126, 127, 132). PDA Tregs express elevated FOXP3 compared to Tregs in the spleen, suggesting PDA-specific antigen recognition, as FOXP3 can be upregulated after TCR signaling (45, 126). Neoadjuvant therapy reduces the frequency of intratumoral Tregs, while increasing in CD8 T cells (129). In addition to Tregs, intratumoral DCs can induce FOXP3- CD4 T cells that induce peripheral tolerance, and are termed type 1 regulatory cells (Tr1) cells (85). Tr1-related genes correlated with poor prognosis in PDA patients (85). The pleiotropic cytokine TGFβ, produced by Tregs and other immunosuppressive cells, can act directly on effector lymphocytes to impair the anti-tumor immune response. CAFs induce Treg differentiation and IL-10 production (46), while Treg production of TGFβ drives αSMA^+^ myCAF differentiation (132). Soluble TGFβ1 impairs NK cell function in PDA patients (96) and TGFβ signaling restrains CD8 T cell cytotoxicity, as CD8 T cells deficient in TGFβ signaling were more effective at lysing tumor cells in an adoptive transfer model into KC mice (133). Autochthonous *KPC* mice treated with TGFβ blockade and anti-PD-1 had increased survival, increased T cell priming, and greater CD8 T cell intratumoral infiltration and Granzyme B production (133).

Treg depletion during early tumor formation accelerated carcinogenesis and accumulated MDSC and immunosuppressive TAMs in the TME without improving CD8 T cell cytotoxicity. In contrast, Treg depletion in an advanced disease led to tumor regression and increased CD8 T cell activation and cytokine production (127, 132). These data indicate both pro-tumor and anti-tumor effects of Tregs depend on disease stage. Tregs can promote tolerance by preventing DC expression of MHC class II, CD40, and CD86 (132). Overall, Tregs both directly limit T cell function and promote immunosuppressive CAFs and myeloid cells that further hinders the T cell anti-tumor response.

### Regulatory B Cells

Recent studies suggest that B cells in PDA have regulatory functions. In both PDA patients and mouse models, B cell production of IL-35 correlated with reduced T cell tumor infiltration and increased Tregs (90, 134). Interfering with IL-35 increased T cell infiltration and cytokine production while reducing tumor weight and PanIN lesion formation (90, 102, 134). IL-35 neutralization in combination with anti-PD-1 improved mouse survival while increasing intratumoral CD8 T cells and IFNγ in PDA mouse models (134). Administration of a BTK inhibitor also reduced tumor burden and increased CD8 T cell production of IFNγ in an orthotopic mouse model (135). B cell depletion with a cocktail of anti-CD19, anti-B220, and anti-CD22 reduced the number of preinvasive lesions in *KC* mice (102). However, B cell depletion with anti-CD20 failed to reduce tumor burden in autochthonous *KPC* mice, contrasting with above studies performed in an implantable setting (136). Human PDA patients treated with neoadjuvant therapy exhibited a reduction in intratumoral B cells (129), and PDA patients with B cells their tumors had reduced overall survival (102). In contrast, B cells also participate in tertiary lymphoid structure (TLS) formation, which are sites within PDA that contain DCs and participate in T cell priming and regulation of the immune response and are associated with improved survival of PDA patients (45). Thus, the specific role of B cells in PDA is currently unclear and may depend on specific B cell subsets, disease stage, and context which they are studied.

## Strategies to Enhance the Efficacy of Immunotherapies

### Autophagy Inhibition

Pancreatic tumor cells typically express low cell surface MHC class I albeit levels are variable among individual patient tumors and even within the same tumor (45). *KPC* cell lines are also low for cell surface MHC class I, which can be upregulated by exposure to IFNγ (23). Tumor escape caused by impaired antigen presentation or MHC class I expression on tumor cells is often attributed to mutations in the MHC class I pathway (22, 23, 137). A recent discovery described a novel mechanism whereby cell surface MHC class I molecules are degraded by tumor cells *via* autophagy (106). Systemic inhibition of autophagy using chloroquine required dual ICB therapy (anti-PD-1 + anti-CTLA-4) for improved T cell infiltration into orthotopic tumors, yielding a 59% response rate (106). Similarly, dual therapy with an ERK inhibitor and chloroquine inhibited tumor growth and improved survival in a PDA xenograft mouse model (138). Interfering with mutant *Kras* signaling in combination with autophagy inhibition has shown objective responses in advanced human PDA (139) though the relative contribution of the immune response remains to be investigated. Thus, autophagy of MHC class I on tumor cells contributes to tumor escape, and autophagy inhibition renders tumors responsive to immunotherapy.

### Agonistic Anti-CD40

CD40 agonist promotes the antitumor activity of a variety of immune-based therapeutic strategies and may do so by engaging DCs, promoting anti-tumor TAMs, and creating a TME that is conducive to anti-tumor cytolytic effector T cells. CD40 agonist altered the cytokines that intratumoral myeloid cells produce, including IL-27, which in turn impacted the intratumoral differentiation of potent cytolytic effector T cells at the expense of exhausted T cells in the TME (64). Agonistic anti-CD40 increased antigen presenting cell antigen processing and presentation to prime and activate antigen-specific T cells, yielding successful anti-tumor responses as a monotherapy or a component of combination approaches (140–142). Treatment of mice bearing subcutaneous tumors with CD40 agonist, anti-PD-1, and anti-CTLA-4 enhanced priming of T cells with unknown antigen-specificity and intratumoral T cell production of TNFα and IFNγ (41).

CCL5 is produced by monocytes, macrophages, cDC2s, and ILC2s to recruit cDC1s to the TME, and intratumoral myeloid cells increased their secretion of CCL5 in response to agonist anti-CD40 therapy (143, 144). Intratumoral CCL5 mediates T cell trafficking to the TME and is required for the efficacy of CD40 agonist + anti-PD-1 + anti-CTLA-4 triple therapy (143). T cells were required for the efficacy of this combination (41, 52). Combination of a TLR4 agonist, agonistic anti-CD40, and anti-PD-1 improved the survival of mice bearing orthotopic tumors, and studies with additional tumor models suggest that this therapy promotes DC priming of naïve T cells and recruitment of antigen-specific T cells to the TME (145). Flt3L administration mobilizes DCs and when combined with agonistic anti-CD40 improved the survival of autochthonous KPPC-OG mice (﻿﻿p48-Cre;Kras^LSL-G12D^;Trp53^fl/fl^;R26^tm1(LSL-OG)^) or mice bearing subcutaneous tumors through the intratumoral enrichment of cDC1s and infiltration of OVA-specific CD8 T cells (56, 108).

A MEK1/2 inhibitor was successful as a single agent in treating mice bearing subcutaneous tumors and synergized with agonistic anti-CD40 to increase mouse survival and prompt rejection in 32% of tumors (146). MEK1/2 inhibitor plus agonistic anti-CD40 dual therapy increased the ratio of intratumoral CD8 T cells to Tregs and increased the frequency of inflammatory anti-tumor macrophages at the loss of immunosuppressive TAMs, whereas agonistic anti-CD40 alone increased CD8 T cell frequency and production of TNFα and IFNγ while also improving DC activation and antigen presentation (146). The MEK1/2 inhibitor alone directly induced tumor cell death, decreased the frequency of intratumoral Tregs and TAMs, yet selectively targeted immunosuppressive TAMs and MDSCs while sparing inflammatory TAMs (146). Similar results were seen with a triple therapy of MEK inhibitor, CDK4/6 inhibitor, and anti-PD-L1 also in a subcutaneous model; triple therapy improved DC antigen presentation, shifted the TME immune landscape to contain more lymphoid cells and fewer myeloid cells as well as enhanced anti-tumor macrophages, CD8 T cells and NK cells (147). These pro-inflammatory anti-tumor TAMs were defined by induction of genes associated with iron metabolism, including *Ftl1* and *Fth1*, as well as genes associated with immune activation including *Bnip3l* and *Ctsd (*147). Overall, agonist anti-CD40 improves T cell functionality and synergizes with other immunotherapies to improve the T cell anti-tumor response.

### Targeting TAMs

Treatment of orthotopic tumor bearing mice with a CD11b agonist (ADH-503) decreased the total number of intratumoral myeloid cells and remaining TAMs exhibited an inflammatory phenotype that promoted intratumoral antigen-specific T cell recruitment and function (103). Autochthonous *KPC* mice treated with CD11b agonist combined with anti-PD-1, anti-CTLA-4, and gemcitabine had significantly improved survival and increased intratumoral CD8 T cell infiltration, but the tumors were not rejected as seen with orthotopic tumor bearing mice (103). However, because *KPC* mice are an autochthonous model, tumor cells are continuously regenerative making curative therapy notoriously difficult. Similarly, genetic ablation of CD11b myeloid cells in an inducible *KC* mouse model led to a reduction in Tregs and PD-L1 expression along with concurrent activation of CD8 T cells in the TME and delayed PanIN lesion formation (17).

Other strategies to overcome TAM suppression specifically target the signaling pathways contributing to their suppressive nature. Blockade of Csf1/Csf1R, which is expressed by some TAMs, improved mouse survival and reduced tumor weight (104). Csf1/Csf1R blockade reduced collagen deposits, αSMA expression by fibroblasts, and congruently increased CD8 T cell infiltration in PDA mouse models (104, 118). Further, TAM depletion reduced immunosuppressive IL-6 and IL-10 and restored CD8 T cell production of Granzyme B, IFNγ, and perforin, and improved survival of autochthonous *KPC* mice (104). In a metastatic *KPC* mouse model, anti-PD-1 and anti-Csf1 synergized to reduce collagen deposits and αSMA+ myofibroblasts, both of which improved CD8 T cell infiltration of PDA (118). Additionally, TAMs in human and murine PDA express PI3Kγ, and genetic ablation or pharmacological inhibition of PI3Kγ reduced tumor weight and metastasis in orthotopic and autochthonous murine models (148). Mechanistically, inhibition of PI3Kγ decreased TAM expression of genes associated with immune suppression and tumor angiogenesis (*Arg1, Tgfb, Il1b, Il6, Vegfa*), and upregulated expression of genes associated with anti-tumor immunity (*Il12 and Ifng*), suggesting TAM reprogramming (148).

### Engineered T Cell Therapies

A promising immunotherapy is adoptive T cell therapy, which includes the isolation, engineering, and expansion of a defined tumor-reactive T cell population for infusion back into patients (149). This approach may be particularly useful in malignancies where there is a low TMB, and thus a robust endogenous anti-tumor response is lacking. Adoptive transfer of large numbers T cells that express a tumor-reactive TCR have demonstrated proof-of principle for tumor eradication in the clinic in melanoma, leukemia, and some solid tumors (150–160). Such transfer of native or engineered TCR-expressing T cells is distinct from chimeric antigen receptor (CAR)-modified T cells, which recognize cell surface tumor antigens independent of MHC molecules (161). CD19 CAR therapies are effective at eradicating some hematological malignancies (162). However, CAR T cell therapies have not been successful on their own in most solid tumor settings. Phase 1 clinical trials of mesothelin-specific CAR T cells demonstrated safety of the therapy but show limited clinical efficacy and poor persistence (163, 164). Efforts to improve the efficacy of mesothelin-specific CAR T cells include the addition of an oncolytic adenovirus that induced TME expression of TNFα and IL-2 (165). This combination therapy vastly improved *in vitro* tumor cell lysis and tumor regression in a xenograft NSG mouse model (165). Addition of TNFα and IL-2 enhanced CAR T cell activation as measured by CD69 and CD25 expression along with proliferation, infiltration, and accumulation in tumors of NSG mice (165). Co-infusion of Msln CAR T cells and CD19 CAR T cells successfully depleted B cells yet failed to improve the persistence of mesothelin-specific CAR T cells (166). Prostate stem cell antigen (PSCA)-specific CAR T cells were modified so that the TGFβ receptor was linked to a stimulatory 4-1BB domain and the IL-4 receptor was linked to IL-7 receptor signaling, switching suppressive TGFβ and IL-4 to stimulatory and survival signals (167). Adoptive transfer of these modified CAR T cells yielded complete rejection of subcutaneous tumors in NSG mice, and tumor re-challenge induced CAR T cell expansion and tumor rejection (167).

TCR engineered CD8 T cells specific to mesothelin have yet to be tested in the clinic. The preclinical studies are promising and although engineered T cells are ultimately rendered exhausted in the TME, serial Msln TCR-engineered T cell infusions in concert with a vaccine to expand infused T cells induced objective responses and doubled survival in the aggressive autochthonous *KPC* animal model (53). We found that administration of the vaccine at the time of T cell infusions was critical for promoting T cell expansion, highlighting the fact that Msln is not presented in an immunogenic manner. However, serial T cell infusions in the clinic are laborious and expensive. Therefore, a major effort by our lab is to identify the factors in the TME that are interfering with engineered T cell function. Notably, accumulating evidence by our lab indicates that strategies to modify the suppressive TME that enhance the activity of endogenous T cells may not yield the same results for infused engineered T cells (23, 53, 114). Engineered T cells are programmed to have a single and defined antigen-specificity and are artificially activated and expanded with anti-CD3/anti-CD28 beads and cytokines *in vitro* prior to transfer, thereby bypassing the requirement for priming *in vivo*. Abrogating TAMs using Csf1R blockade had no benefit for engineered T cell accumulation or function (114). In contrast, depleting Csf1R+ TAMs increased endogenous T cell infiltration and responsiveness to PD-1 blockade in an animal model in which antigen-specificity of the T cells is unknown (118). One approach that produced beneficial results with both endogenous (38, 64) and engineered T cells was agonistic anti-CD40 (114). However, while agonist anti-CD40 supported cytotoxic activity of endogenous tumor-specific T cells (64), it failed to rescue cytotoxic function and cytokine production of infused engineered T cells (114). As agonistic anti-CD40 alone is suboptimal in priming and expanding endogenous antigen-specific T cells (64, 168), future combinations with TLR agonists seem promising as this has been shown to dramatically improve vaccination (168). Thus, ICB which was assumed to rescue intratumoral T cell function, may instead act systemically on a new wave of recently activated T cells (23), which are not present in the engineered T cell infused population. Thus, we continue to be cautious to extrapolate results based on endogenous T cell responses to engineered T cell responses.

Of note, the majority of the tumor mass in PDA is stromal cells rather than tumor cells (2). In animal models, MHC class I expression on stromal cells is required for T cell-mediated solid tumor eradication (169). Cross-presentation of tumor antigen by cDC1s that express the transcription factor Batf3 induce priming of rare naïve tumor-reactive T cells (170). Based on data from *Batf3^-/-^* mice, cDC1s appear to also be required for adoptively transferred T cells to infiltrate melanoma (171) and for islet infiltration by islet-reactive T cells (172). This suggests a role for cDC1 antigen presentation in the draining lymph node and/or within tumors for T cell migration into tissues and is consistent with clinical data where tumors with high numbers of cDC1s bode better for immunotherapy outcomes (56, 173, 174). Insufficiency of cDC1s and priming of antigen specific T cells is a major road-block to effective immunotherapies, suggesting a need for therapies that draw antigen-specific T cells into the TME and also maintain T cell activation within the TME. cDC1 production of CXCL9 and CXCL10 recruits T cell into the TME (103, 171). Moreover, in a MC38 tumor model, response to anti-PD-1 was dependent on intratumoral cDC1 production of CXCL9 and subsequent signaling through CXCR3 on T cells, which promoted proliferation and production of IFNγ, TNFα, and Granzyme B (175). In a breast cancer model, expansion of adoptively transferred tumor antigen-specific T cells was mediated by DC engagement through a combination therapy of Flt3L, radiotherapy, poly(I:C), and a CD40 agonist (176). Adoptive transfer of CAR T cells engineered to secrete Flt3L and in combination with poly(I:C) and anti-4-1BB enhanced T cell and DC expansion and activation in various tumor models (177). Thus, unlike CARs, TCRs provide T cells the ability to recognize peptide:MHC complexes not only on the tumor cells, but also on antigen presenting cells such as cDC1s, which may be a particular advantage of TCR based approach over CAR-based engineering strategies (53, 114).

### Immune Checkpoint Blockade

Similar to PDA patients, anti-PD-L1 or anti-PD-1 monotherapy is ineffective in *KPC* mouse models and provides minimal benefit in survival or tumor regression even in animal models with a model neoantigen (23, 82, 83, 87, 90, 101, 103, 118, 133, 134). Both TMB and T cell inflamed gene expression profile are biomarkers for response to immune checkpoint blockade (ICB) in human PDA and other cancers (178). Although a low TMB in PDA may partially explain resistance to ICB, it is clear based on trials with MSI^high^ PDA that this is not the full story (34).

While anti-PD-1 therapy alone is not successful at treating PDA, it synergizes with many other immunotherapies in preclinical studies and thus we are hopeful that in the near future combinations will prove efficacious in the clinic (37). We identified that anti-PD-1 plus anti-PD-L1 treatment of mice bearing orthotopic tumors improved mouse survival and induced systemic expansion of tumor-specific T cells that were then recruited to the TME and further differentiated into central and tissue resident memory T cells following orthotopic tumor clearance (23). Importantly, dual therapy promoted Klrg1+ effector cells producing IFNγ and TNFα and decreased the frequency of PD-1 and Lag3 expressing T cells due to recruitment of new effector cells and not reinvigoration of intratumoral exhausted T cell (23). These results raise the question if reinvigoration of intratumoral T cells is possible in PDA. Dual therapy with anti-PD-1 + anti-OX40 led to complete response and long-term survival along with recall response to re-challenge in an orthotopic tumor model, while autochthonous *KPC* mice had improved survival by 40 days (83). This dual therapy required CD4 T cells for efficacy and memory response, and led to intratumoral enrichment of total CD4 T cells, CD8 and CD4 memory T cells, and a reduced frequency of Tregs and PD-1+ T cells (83). Similarly, anti-PD-1 + anti-OX40 improved the efficacy of PancVAX, a neoantigen targeted vaccine with a STING adjuvant, and induced a durable response in mice bearing subcutaneous tumors from a Panc02 cells line and that correlated with reduced frequency of intratumoral T cells co-expressing PD-1 and Lag3 (179). However this dual therapy was not tested without the vaccine and may have been the main therapeutic driver (179). In orthotopic but not subcutaneous mouse models, IL-33 activates ILC2s, which in turn produce CCL5 to recruit CD103+ cDC1s to the TME which then prime and activate CD8 T cells to infiltrate the tumor and exert an anti-tumor immune response (144). Dual therapy with recombinant IL-33 plus anti-PD-1 engaged ILC2s to accumulate in orthotopic *KPC* tumors and improved T cell infiltration through DC priming, allowing for improved mouse survival and decreased tumor volume (144). Overall, anti-PD-1 therapy synergizes with many other immunotherapy drugs to improve the anti-tumor immune response of T cells in PDA mouse models. The challenge ahead is to identify the approaches that can be safely used in patients and that elicit durable responses.

## Challenges to Modeling the Immune Response in PDA Preclinical Studies

Successful immunotherapy for targeting PDA will likely require multiple agents used in combination and sequenced appropriately. Target identification as well as safety and efficacy testing require rational immune-competent preclinical animal models. There are many labs utilizing variations of the *KPC* animal model. How this model is used to interrogate immunotherapy response is quite important, however. In the autochthonous setting, high-resolution ultrasound imaging should be performed to locate and measure a defined tumor mass within the pancreas to ensure that mice have invasive disease before enrolling for treatments or else regimens will be tested in animals with only preinvasive disease. Implanted *KPC* cell lines under the skin of wild type animals will likely not fully recapitulate the TME of the native organ as most of the myeloid cells in the skin tumors will be of blood origin and such tumors will lack pancreas tissue resident macrophages (113). Orthotopic *KPC* tumor implantation is a better alternative to subcutaneous tumors. As discussed, a major limitation is the lack of identified native antigens in the *KPC* model to interrogate endogenous tumor-specific T cells. While Ova can be used, as tumors that express Ova are rejected, it remains questionable the extent this models a T cell response in PDA. We target mesothelin with engineered T cells, but we also showed that most endogenous T cells reactive to Msln are deleted during T cell development (53). Thus, while we developed a neoantigen model by engineering the tumors to express CB as discussed above, the epitope binds MHC with high affinity and thus models a highly immunogenic epitope. Consequently, there remains a major knowledge gap regarding the specificity of the CD4 and CD8 T cells that are engaged during combination therapies and the extent that the epitopes are immunogenic on their own.

## Summary

Mechanistic studies support that priming and expanding systemic T cells, rather than rescuing or reinvigorating exhausted T cells, is necessary for tumor rejection. This is in opposition to the preponderance of the literature describing T cell exhaustion during chronic viral infection. There are striking differences between chronic viral infection and PDA including the location of the target antigen and the surrounding environment. Chronic LCMV results in a systemic infection where antigen and antigen presentation are not limiting, and the biology of the T cells is almost always assessed from the spleen. In contrast, many studies support that antigen and/or antigen presentation is limiting in PDA and thus T cell differentiation toward the identical exhausted T cell state is unlikely, and lack of T cell function may be due to the myriad of suppressive mechanisms described above.

**Figure 3:**
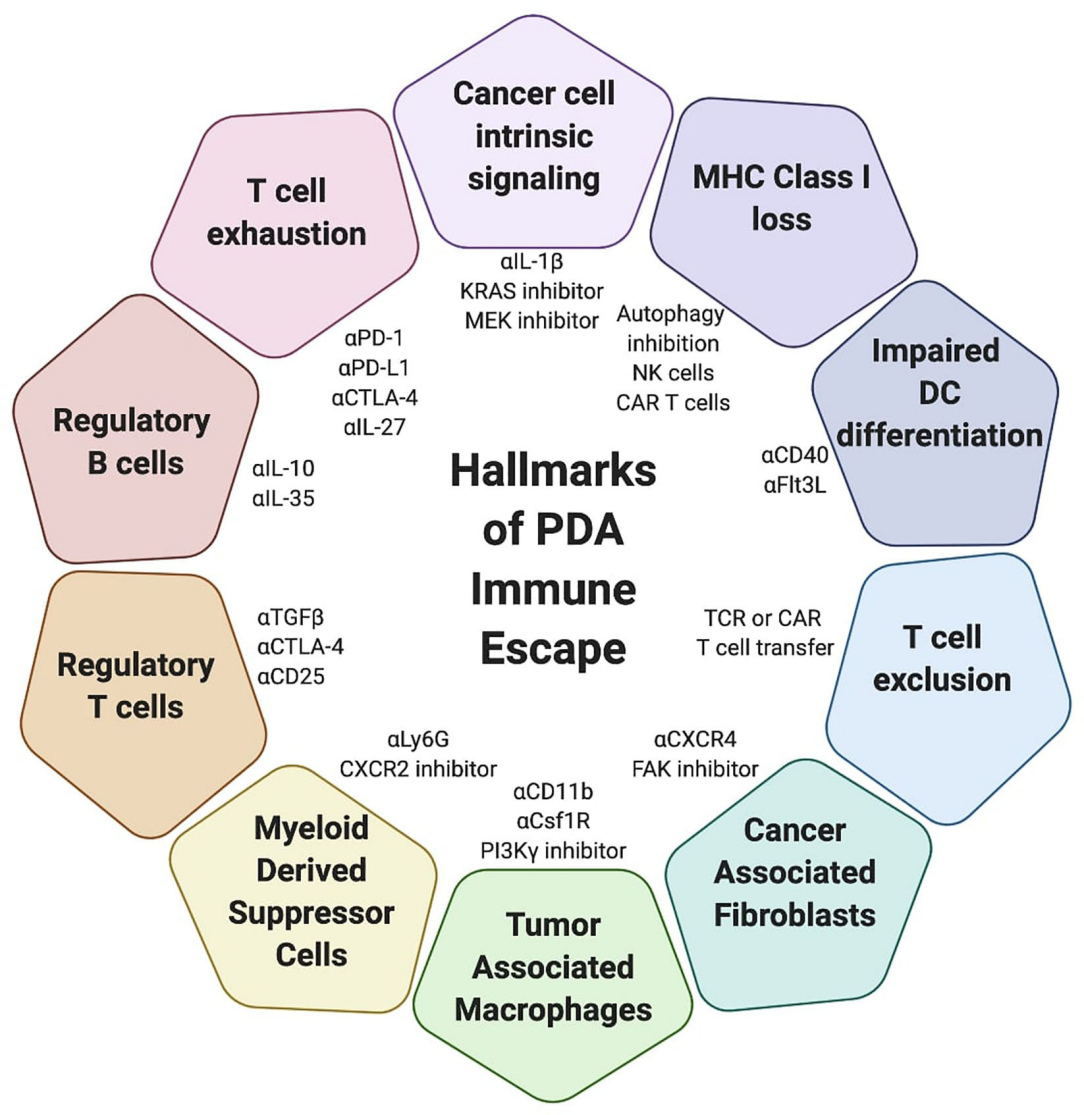
Mechanisms of immune escape in pancreatic cancer. Simplified overview of mechanisms by which pancreatic ductal adenocarcinoma (PDA) evades the immune response and examples of potential therapeutic strategies to target these mechanisms. Some strategies may obviate multiple tumor evasion mechanisms. Combination strategies may be necessary to promote synergy and overcome tumor evasion from the immune system.

PDA is characterized by a dense desmoplastic stroma and enrichment of immunosuppressive cells. While antigen-specific lymphocytes can infiltrate the TME, proliferate and mediate some anti-tumor activity transiently, they lose function either through progressive exhaustion due to chronic TCR signaling and/or immunosuppressive cells and cytokines that limit their activation and cytotoxicity. PDA is resistant to single agent T cell-directed immunotherapies, and combination therapies targeting lymphocytes and reprograming the TME are necessary to induce a productive anti-tumor immune response. While there are many combination therapies that induce a partial or complete response in murine models, clinical trials have yet to successfully identify an effective immunotherapy to treat PDA patients. As such, further investigation into the individual mechanisms that limit T cell function in PDA is required. Moreover, these individual mechanisms likely form a hierarchy of suppression. By elucidating the key mechanism(s) of suppression we can identify the best targets for PDA immunotherapy under specific conditions of T cell activation, suppression or exhaustion (**Figure 3**). For example, immune checkpoint blockade may complement approaches that can engage tumor-specific T cells while simultaneously modifying the immunosuppressive TME. A deficiency in T cell priming can may be addressed by approaches that expand and activate DCs or adoptive T cell therapy. However, once a sufficient T cell response is engaged, if tumor cells are not eradicated, tumor escape either through defects in target antigen and/or antigen presentation, or T cell exhaustion due to chronic antigen encounter may prevail. Thus, identifying strategies to anticipate and overcome escape following immunotherapy may be critical for durable responses in the clinic.

## Author Contributions

All authors contributed to the article and approved the submitted version.

## Funding

IS is supported by an AACR Pancreatic Cancer Action Network Career Development Award (17-20-25-STRO), AACR Pancreatic Cancer Action Network Catalyst Award (19-35-STRO), an American Cancer Society Institutional Research Grant (124166-IRG-58-001-55-IRG65), and pilot awards from the Masonic Cancer Center and Cancer Research Translational Initiative, (University of Minnesota Medical School) and NIH U54-CA-210190.

## Conflict of Interest

IS serves on the scientific advisory board for Luminary Therapeutics and Immunogenesis.

The remaining author declares that the research was conducted in the absence of any commercial or financial relationships that could be construed as a potential conflict of interest.
